# Prognostic Value of Very Early Interim FDG PET/CT After Single Cycle of Chemotherapy for 10-Year Survival in Diffuse Large B-Cell Lymphoma

**DOI:** 10.3390/cancers17060926

**Published:** 2025-03-08

**Authors:** Eun Ji Han, Hye Lim Park, Seung-Ah Yahng, Gi-June Min, Byung-Ock Choi, Gyeongsin Park, Joo Hyun O, Seok-Goo Cho

**Affiliations:** 1 Division of Nuclear Medicine, Department of Radiology, Yeouido St. Mary’s Hospital, College of Medicine, The Catholic University of Korea, Seoul 06591, Republic of Korea; iwao@catholic.ac.kr; 2 Division of Nuclear Medicine, Department of Radiology, Eunpyeong St. Mary’s Hospital, College of Medicine, The Catholic University of Korea, Seoul 06591, Republic of Korea; prhlim@gmail.com; 3 Department of Hematology, Incheon St. Mary’s Hospital, College of Medicine, The Catholic University of Korea, Seoul 06591, Republic of Korea; saymd@catholic.ac.kr; 4 Department of Hematology, Seoul St. Mary’s Hospital, College of Medicine, The Catholic University of Korea, Seoul 06591, Republic of Korea; beichest@catholic.ac.kr; 5 Department of Radiation Oncology, Seoul St. Mary’s Hospital, College of Medicine, The Catholic University of Korea, Seoul 06591, Republic of Korea; choibo67@catholic.ac.kr; 6 Department of Hospital Pathology, Seoul St. Mary’s Hospital, College of Medicine, The Catholic University of Korea, Seoul 06591, Republic of Korea; gspark@catholic.ac.kr; 7 Division of Nuclear Medicine, Department of Radiology, Seoul St. Mary’s Hospital, College of Medicine, The Catholic University of Korea, Seoul 06591, Republic of Korea

**Keywords:** ^18^F-FDG PET/CT, DLBCL, early interim, long-term, outcome

## Abstract

Background/Objectives This study aimed to evaluate whether very early interim ^18^F-fluoro-2-deoxyglucose (FDG) positron emission tomography (PET)/computed tomography (CT) after a single cycle of first-line chemotherapy predicts long-term survival outcome in patients with diffuse large B-cell lymphoma (DLBCL). Methods A total of 51 patients (31 males and 20 females; mean age 55 years) had four FDG PET/CT studies, at baseline and after one, three, and six cycles of chemotherapy (PET0, PET1, PET3, and PET6). Visually and quantitatively assessed PET parameters were analyzed for associations with long-term survival. Results The estimated 10-year progression-free survival (PFS) and overall survival (OS) was 48% and 61%, respectively. During a median follow-up of 63 months (range 9–134), 17 patients (33%) exhibited disease progression and 15 (29%) died. On PET1, all but one showed decreased FDG uptake, and all showed decreased metabolic tumor volume. None of the PET1 or PET3 parameters were associated with survival. The PET6 parameters retained independent predictive value for OS after adjustment for the International Prognostic Index. Negative PET6 was associated with longer PFS (mean 99 vs. 50 mo, *p* = 0.04) and OS (mean 107 vs. 57 mo, *p* = 0.02). Con-clusions The FDG PET/CT parameters obtained after a single cycle of chemotherapy were not associated with long-term survival in DLBCL, while negative end-of-therapy FDG PET/CT was associated with longer PFS and OS. Tumor regression very early into first-line chemotherapy was not as clinically relevant as the presence of viable tumor on FDG PET/CT at the end of therapy for predicting long-term outcomes.

## Simple Summary

Through FDG PET/CT obtained at four different time points, lymphoma lesions were followed during first-line therapy and FDG PET/CT had prognostic value for long-term survival at 10 years. The rate of tumor regression very early into first-line chemotherapy was not as relevant as the presence of viable tumor on FDG PET/CT at the end of therapy for predicting long-term outcomes.

## 1. Introduction

Diffuse large B-cell lymphoma (DLBCL) is well known to be the most common subtype of non-Hodgkin’s lymphoma (NHL), accounting for about 30% of all cases, and is clinically a very heterogeneous disease. Rituximab plus cyclophosphamide, hydroxydaunomycin, vincristine, and prednisone (R-CHOP) has been the standard first-line treatment for patients with DLBCL for nearly two decades; approximately one third of the patients will not respond to this treatment and have aggressive clinical courses [1,2]. Accurate risk stratification of patients can help to predict outcomes and support tailored treatment strategies. The International Prognostic Index (IPI) is used at baseline for risk stratification of patients with DLBCL and takes into account age, Eastern Cooperative Oncology Group (ECOG) performance status, lymphoma stage, serum lactate dehydrogenase (LDH), and number of extranodal sites [3].

Currently, ^18^F-fluoro-2-deoxyglucose (FDG) positron emission tomography (PET)/computed tomography (CT) is considered essential at the baseline and the end of first-line therapy in most lymphoma [4]. In addition, interim FDG PET/CT is a crucial imaging tool for predicting therapeutic outcomes and response-adapted therapy in most patients with Hodgkin’s lymphoma (HL) [5,6]. Although current guidelines carefully recommend interim FDG PET/CT in patients with DLBCL due to the possibility of false positives, interim FDG PET/CT is already actively performed in the research setting and clinical practice [4,7,8]. In DLBCL, interim FDG PET/CT is recommended after 2–4 cycles of R-CHOP, and the optimal timing has not been fully investigated. Metabolic decline in lymphoma lesions occurs immediately and rapidly after the initiation of chemotherapy [9,10]. This leads to the possibility that very early interim FDG PET/CT after one cycle of R-CHOP can be used to tailor the treatment approaches for patients with DLBCL. In our previous results [11], a good response in interim FDG PET/CT after one cycle of R-CHOP was correlated with complete metabolic response (CMR) at the end of therapy, but did not predict one-year disease-free status.

Therefore, we performed this study to evaluate whether very early interim FDG PET/CT after a single cycle of R-CHOP chemotherapy has prognostic value independent of IPI score and improves the prediction of long-term survival outcome in patients with DLBCL. We also explored the metabolic kinetics to R-CHOP through FDG PET/CT obtained at four different time points.

## 2. Materials and Methods

### 2.1. Study Design and Patients

A cohort prospectively enrolled at a tertiary hospital specializing in hematologic malignancies between March 2012 and December 2017 (ClinicalTrials.gov identifier NCT01357733) was retrospectively reviewed and analyzed for this study. The objective of the prospective study was to evaluate whether very early response assessment by PET/CT after one cycle of R-CHOP is predictive of treatment success in patients with DLBCL, and the results in this study population have been reported in a previous publication [11]. Consecutive adult patients (>18 years) with confirmed CD20-positive DLBCL and no prior treatment for lymphoma were enrolled in the study. Exclusion criteria were pregnancy and primary central nerve system lymphoma. All patients received R-CHOP chemotherapy as the standard first-line treatment and underwent four FDG PET/CT studies using the same predetermined protocol and system: at baseline (PET0), and after one (PET1), three (PET3), and six cycles of chemotherapy (PET6). PET1 was performed 2 to 48 h before the second cycle of R-CHOP was administered. The PET1 findings were not used to change the course of therapy. After the completion of R-CHOP chemotherapy, regular clinical follow-up included physical examination, performance status, and routine blood lab examination every 3 months, and contrast enhanced CT of the neck, chest, and abdomen every 3 or 6 months.

The study was approved by the institutional review board of our hospital (KC11EISI0293). All patients provided written informed consent in accordance with institutional guidelines.

### 2.2. FDG PET/CT Acquisition

Fasting for at least 6 h was mandatory before FDG 5.2–7.8 MBq/kg was injected intravenously, and scanning began strictly 60 min later. None had blood glucose levels > 180 mg/dL. Biograph Truepoint (Siemens Medical Solutions, Knoxville, TN, USA) was used for all patients. Non-contrast enhanced CT from the vertex and to the thighs was obtained (120 kVp, 80 mAs, 3-mm slice thickness) and PET followed immediately (1.5–2.5 min per bed position). The CT data were used for attenuation correction, and PET images were reconstructed using standard-ordered-subset expectation maximization (two iterations, eight subsets). The axial spatial intrinsic resolution of the system was 4.2 mm at the center of the field of view.

### 2.3. FDG PET/CT Image Analysis

Two experienced nuclear medicine physicians unaware of patient information reviewed all FDG PET/CT images using XD3 (Mirada Medical, Oxford, UK). Response was visually assessed using the Deauville 5-point scale: Deauville score (DS) 1, no uptake; score 2, uptake ≤ mediastinum; score 3, mediastinum < uptake < liver; score 4, uptake moderately > liver; and score 5, uptake markedly > liver or any new lesion [4]. DS 1, 2, and 3 were considered PET-negative and DS 4 and 5 PET-positive. If there was disagreement between the readers, consensus was achieved via review by a third nuclear medicine physician. In the quantitative analysis, peak standardized uptake value corrected for lean body mass (SULpeak) was derived semi-automatically using a spherical volume of interest (VOI) of 1 cm^3^ [12]. We also measured the metabolic tumor volume (MTV) of all lymphoma lesions, and average of 1.5 × SUL + 2 standard deviation (SD) in the liver was used to define each threshold. The percent changes in SULpeak and MTV at early interim between PET0 and PET1 (%ΔSUL1 and %ΔMTV1), at interim between PET0 and PET3 (%ΔSUL3 and %ΔMTV3), and at end of therapy between PET0 and PET6 (%ΔSUL6 and %ΔMTV6) were calculated. SULpeak and MTV were measured independently, and the measurements were repeated when the numbers were not equal.

### 2.4. Statistical Analysis

The primary endpoint was overall survival (OS), which was defined as the time from the date of PET0 to the date of death from any cause or last clinical follow-up. Progression-free survival (PFS) was defined as the time from the date of PET0 to the date of detected disease progression/recurrence or death. The Kaplan–Meier method was used to estimate survival times, and the log-rank test was used for assessment of survival differences between groups. Univariate Cox proportional hazards regression was used to identify prognostic factors for PFS and OS. Based on the results of the univariate analysis, different multivariate Cox proportional hazards models were used to evaluate the independent prognostic effect of each PET parameter after adjustment for the effects of significant clinical factors. All statistical analyses were performed using SPSS version 26 (IBM Corp., Armonk, NY, USA). Differences with *p* value < 0.05 were considered statistically significant.

## 3. Results

### 3.1. Patient Characteristics

Of the 54 patients enrolled, one patient was excluded due to change in therapeutic regimen with disease progression after three cycles of R-CHOP, and two patients chose to drop out before completion of chemotherapy. Therefore, a total of 51 consecutive patients with DLBCL treated with full cycles of R-CHOP chemotherapy combined with four FDG PET/CT studies were included in this study. All imaging was performed under the same predetermined imaging conditions using same scanner. At baseline, 27 patients (53%) had an IPI score of 0–2 and 24 (47%) had a high IPI score of 3–5 (Table 1). The therapy following first-line R-CHOP chemotherapy varied among the patients. Of the 51 patients, six (12%) who achieved remission following first-line chemotherapy had autologous stem cell transplantation (SCT); two (4%) had allogeneic SCT for nonresponding disease; and 33 (65%) received first-line R-CHOP chemotherapy only. During a median follow-up of 63 months (range 9–134 months), disease progression was seen in 17 patients (33%) and death due to any cause occurred in 15 patients (29%). Four patients experienced disease progression more than 5 years after first-line chemotherapy. Of the 17 patients with progression, two remained alive after salvage chemotherapy (at the time of publication time from baseline, 109 months and 130 months). The estimated 1-, 2-, 5-, and 10-year PFS rates were 88%, 78%, 75%, and 48%; and OS rates were 96%, 84%, 75%, and 61%, respectively.

### 3.2. Early Interim Response

**Figure 1:**
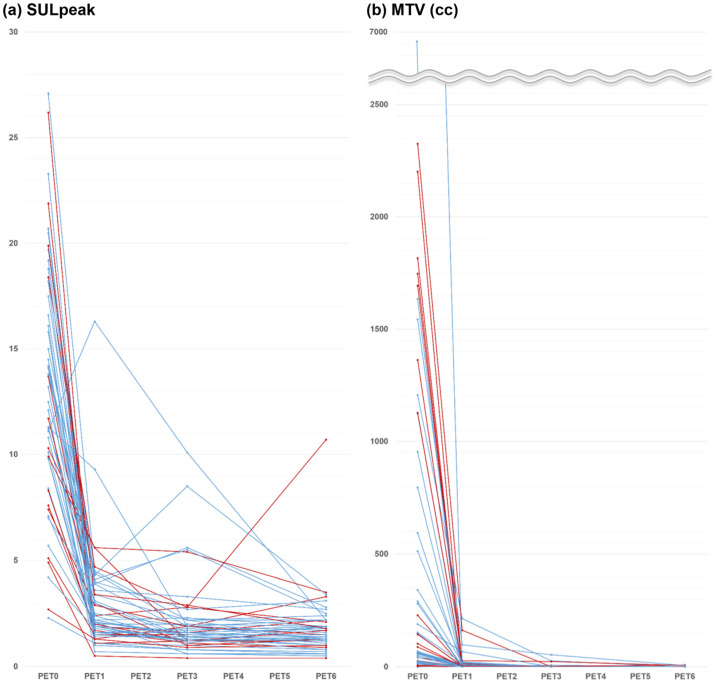
Periodic changes of SULpeak (**a**) and MTV (**b**) for each of 51 patients (survival denoted by blue and death denoted by red).

Early PET assessment following a single cycle of R-CHOP was PET1-negative in 27 patients (53%) and PET1-positive in 24 (47%). On quantitative assessment of PET1, all but one patient showed decreased SULpeak and all patients showed decreased MTV (Figure 1). Details are available in a previous publication exploring early interim response evaluation [11].

### 3.3. Survival Analysis

**Figure 2:**
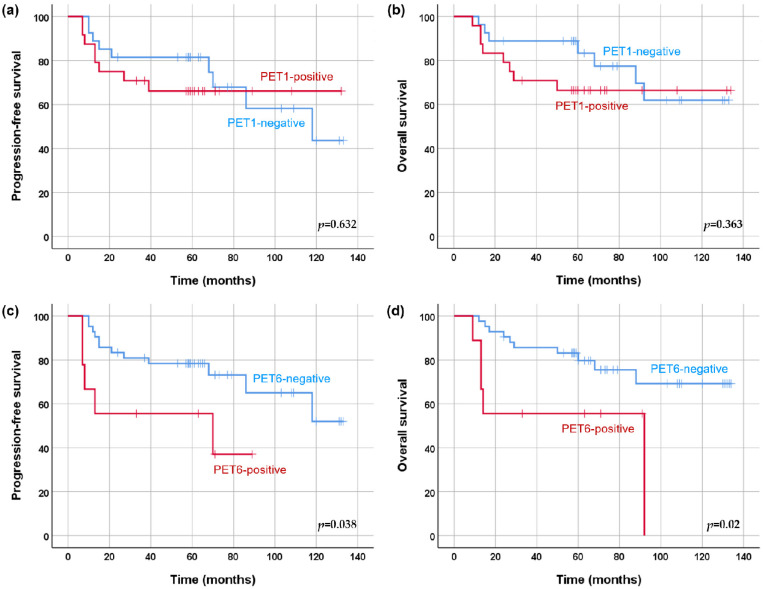
PFS and OS according to FDG PET response after one (**a**,**b**) and six cycles (**c**,**d**) of R-CHOP chemotherapy.

In univariate analysis, a high IPI score was a risk factor for PFS (*p* = 0.03), but not for OS (*p* = 0.062) (Table 2). None of the PET1 or PET3 parameters were significantly associated with survival. All PET6 parameters were significantly associated with OS (Table 3). Based on the results of the univariate analysis, each multivariate Cox proportional hazards model was established from the IPI score and the PET6 parameters. SULpeak and MTV at PET6 remained as significant predictors of PFS after adjustment for IPI score in each analysis. All five PET6 parameters were tested one at a time against OS controlling for IPI score, and the effect remained significant in multivariate analyses. The patients with PET1-negative results did not have significantly different PFS and OS compared to PET1-positive patients (mean PFS, 95 months versus 93 months; mean OS, 105 months versus 97 months). PFS and OS were significantly longer in patients with PET6-negative results than in those with PET6-positive results (mean PFS, 99 months versus 50 months, *p* = 0.038; mean OS, 107 months versus 57 months, *p* = 0.02) (Figure 2). Of eight patients with MTV > 0 cc (range 0.1–8.1) at PET6, five (63%) experienced disease progression and subsequently died. Of 43 patients with MTV of 0 cc at PET6, 12 (24%) experienced disease progression and 10 (23%) died.

## 4. Discussion

The prognostic value of very early interim FDG PET/CT following only a single cycle of first-line R-CHOP chemotherapy was evaluated for long-term outcomes in patients with DLBCL. Our results demonstrated that none of the FDG PET parameters obtained at this early time point were associated with long-term survival outcomes, while FDG PET parameters obtained at the end-of-therapy had significant value in predicting PFS and OS. The rates of decline or growth of tumor illustrated through FDG PET obtained at different time points may represent an aspect of tumor biology that could be missed on single time-point assessment [13]. Thus, FDG PET/CT images were obtained at four different time points under strictly controlled uniform imaging conditions. On PET1, all but one patient showed decreased FDG uptake intensity, and all patients showed decreased tumor volume.

Interim FDG PET can track metabolic changes during treatment and enable early risk stratification in patients of lymphoma. Early identification of chemo-sensitive patients allows a reduction in dose intensity, which can provide satisfactory tumor control and minimize side effects. On one hand, early identification of late or non-responders may provide an opportunity to improve poor prognosis by dose escalation or modification of the treatment plan [14,15]. Many studies have reported predictive and prognostic values of interim PET in DLBCL [16]. Previous prospective studies showed that interim PET-negative results after two cycles of R-CHOP were significantly associated with treatment success in patients with DLBCL, similar to our previous results [11,17,18]. In this study, neither interim PET/CT after one cycle of R-CHOP nor interim PET/CT after three cycles was associated with long-term outcomes in patients with DLBCL, supporting previous studies that reported the lack of predictive value of interim PET/CT [19]. However, a few studies reported that interim PET-positive results were significantly associated with worse survival [17,18]. In a prospective study with large sample size (*n* = 609), interim PET after two cycles of R-CHOP predicted 2-year event-free survival and OS independent of the IPI score, but treatment intensification based on interim PET did not change outcomes [20]. The prognostic role of interim PET in predicting survival outcome in patients with DLBCL remains debatable.

We added FDG PET/CT after a single cycle of R-CHOP, under the assumption that very early assessment with a higher volume of remaining tumor may better discriminate response rates compared to a later time point when the remaining tumors may be under the detection limits of PET/CT systems. Interim FDG PET/CT is usually recommended after 2–4 cycles of R-CHOP in DLBCL [7]. A previous study investigating the optimal timing of interim PET in patients with DLBCL showed no difference in prognostic values between PET1 (*n* = 30) and PET after two cycles of R-CHOP (*n* = 30) using both SUV change and DS [21]. Other studies have shown that PET1 could predict short-term outcomes [22,23,24]. However, in a more recent comparative study with a large sample size (*n* = 1692), including interim PET at various time points after 1–4 cycles, PET1 alone failed to discriminate between good and poor responders [25]. To our knowledge, no study has investigated the prognostic value of PET1 for long-term survival outcomes. Our median follow-up was 63 months, and the 10-year PFS and OS rates were 48% and 61%, respectively. After the introduction of R-CHOP in patients with DLCBL, the 10-year OS in the real-world setting is thought to be in the range of 51–66%, similar to our results [26,27]. Relapse most often is within the first 2–3 years after initial treatment, and relapse rarely occurs after more than 5 years [28]. However, in our results, 24% of patients (*n* = 4/17) with disease progression had progression after more than 5 years.

Several retrospective and prospective studies have also shown that end-of-therapy PET is predictive of survival [18,29,30]. In our results, both visual and quantitative PET6 assessments were associated with even longer-term outcomes at 10 years. Although the current guidelines recommend visual analysis in the response assessment of patients with lymphoma [7], quantitative analysis may be more informative and objective. In previous studies in patients with DLBCL, SUV-based analysis at interim PET was a better response criterion than visual analysis based on the Deauville 5-point scale [25,29,31].

The relatively small number of patients is a major limitation of this study. However, our study enrolled a homogeneous set of patients, imposed strictly controlled imaging conditions, and obtained long-term outcomes (median follow-up 63 months and maximum 134 months). All patients in this study received first-line R-CHOP chemotherapy and all FDG PET/CT studies were from same time points under predetermined imaging conditions using the same scanner. Therefore, we were able to analyze the absolute SUV and MTV values without the need for standardization to obtain precise quantitative measurements [32,33]. Another limitation was that we did not investigate total lesion glycolysis, which is reported to be a promising predictive marker [30,34]. Although current guidelines recommend visual analysis for response assessment in patients with lymphoma, standardized volumetric parameters are being promoted as imaging biomarkers in DLBCL and may enter the clinical practice in the near future.

## 5. Conclusions

FDG PET parameters obtained very early after one cycle of first-line R-CHOP chemotherapy were not associated with long-term survival in DLBCL. On the other hand, negative end-of-therapy FDG PET retained its significant association with longer PFS and OS. The rate of tumor regression very early into first-line therapy was not as clinically relevant as the presence of viable tumor at the end of therapy for long-term survival outcome.

## Figures and Tables

**Table 1:** Clinical characteristics of 51 patients.

Variables		No. of Patients
Age (years)	Mean ± SD (range)	55 ± 15 (21–81)
	≤65	30 (59%)
	>65	21 (41%)
Sex	Male	31 (61%)
	Female	20 (39%)
ECOG PS	0–1	40 (78%)
	2–4	11 (22%)
LDH level	Normal	25 (49%)
	Elevated	26 (51%)
Extranodal involvement	0–1	22 (43%)
	≥2	29 (57%)
Ann Arbor stage	I	5 (10%)
	II	16 (31%)
	III	12 (24%)
	IV	18 (35%)
IPI score	0	6 (12%)
	1	11 (21%)
	2	10 (20%)
	3	13 (25%)
	4	8 (16%)
	5	3 (6%)
Histologic subtype	GCB	20 (39%)
	Non-GCB	25 (49%)
	Unknown	6 (12%)

**Table 2:** Univariate analysis of clinical variables for PFS and OS.

Variables	PFS		OS	
	HR (95% CI)	*p* Value	HR (95% CI)	*p* Value
Age (≤65 y vs. >65 y)	3.35 (1.206–9.303)	0.020 *	2.872 (1.018–8.104)	0.046 *
Sex (male vs. female)	0.404 (0.131–1.248)	0.115	0.549 (0.175–1.725)	0.304
ECOG PS (0–1 vs. 2–5)	1.456 (0.466–4.547)	0.518	1.461 (0.465–4.593)	0.516
LDH (normal vs. elevated)	3.978 (1.263–12.532)	0.018 *	3.335 (1.056–10.527)	0.040 *
Extranodal involvement (<2 vs. ≥2)	1.671 (0.615–4.538)	0.314	1.795 (0.612–5.265)	0.287
Stage (I–II vs. III–IV)	3.284 (1.041–10.365)	0.043 *	3.785 (1.056–13.572)	0.041 *
IPI score (0–2 vs. 3–5)	3.271 (1.124–9.518)	0.030 *	2.792 (0.952–8.188)	0.062

* *p* < 0.05.

**Table 3:** Univariate and multivariate analyses of PET parameters for PFS and OS.

		PFS				OS			
		Univariate		Adjusted for IPI		Univariate		Adjusted for IPI	
		HR (95% CI)	*p* Value	HR (95% CI)	*p* Value	HR (95% CI)	*p* Value	HR (95% CI)	*p* Value
PET1	DS	1.26 (0.48–3.31)	0.634			1.60 (0.58–4.46)	0.368		
	SULpeak	0.91 (0.67–1.22)	0.516			0.96 (0.74–1.25)	0.762		
	MTV	1.00 (0.99–1.01)	0.840			1.00 (0.99–1.01)	0.762		
	%ΔSUL1	1.00 (0.98–1.03)	0.767			1.00 (0.98–1.02)	0.891		
	%ΔMTV1	0.99 (0.97–1.01)	0.434			0.99 (0.96–1.01)	0.259		
PET6	DS	2.90 (1.00–8.39)	0.049 *	–		3.34 (1.13–9.82)	0.029 *	3.34 (1.13–9.82)	0.029 *
	SULpeak	1.42 (1.06–1.91)	0.019 *	1.34 (1.00–1.82)	0.054	1.65 (1.14–2.39)	0.008 *	1.65 (1.14–2.39)	0.008 *
	MTV	1.33 (1.07–1.66)	0.011 *	1.33 (1.07–1.66)	0.011 *	1.37 (1.09–1.71)	0.006 *	1.37 (1.09–1.71)	0.006 *
	%ΔSUL6	0.97 (0.93–1.01)	0.113			0.95 (0.91–1.00)	0.029 *	0.94 (0.90–0.99)	0.014 *
	%ΔMTV6	0.37 (0.16–0.86)	0.021 *	–		0.23 (0.09–0.61)	0.003 *	0.24 (0.09–0.61)	0.003 *

* *p* < 0.05.

## Data Availability

The data presented in this study are available upon reasonable request.
